# Sequential Arch-Tear Eligibility for Uncovered Aortic Arch Stent in Acute DeBakey Type I Aortic Dissection

**DOI:** 10.3390/jcdd13100496

**Published:** 2026-10-03

**Authors:** Dario Petrone, Bruno Chiarello, Giulia Bessi, Alan Ciardo, Beatrice Bacchi, Francesco Cabrucci, Aleksander Dokollari, Antonio Rosciano, Edvin Prifti, Massimo Bonacchi

**Affiliations:** 1 Cardiac Surgery F.U., Department of Experimental and Clinical Medicine, University of Florence, 50134 Florence, Italyalan.ciardo@gmail.com (A.C.);; 2 Division of Cardiac Surgery, St. Michael’s Hospital, University of Toronto, Toronto, ON M5B 1W8, Canada; 3 Division of Cardiac Surgery, Peter Munk Cardiac Centre, Toronto General Hospital, University Health Network, Toronto, ON M5G 2C4, Canada; 4 Cardiac Surgery Division, St. Boniface Hospital, University of Manitoba, Winnipeg, MB R2H 2A6, Canada; 5 Cardiac Surgery Division, University Hospital Center of Tirana, 1000 Tirana, Albania

**Keywords:** acute aortic dissection, aortic arch, AMDS, anatomical eligibility, computed tomography, false lumen, distal anastomotic new entry

## Abstract

Background: Because an uncovered arch scaffold does not provide a covered seal across an unresected arch tear, we evaluated a source-adjudicated sequential eligibility framework separating study-defined stop gates from residual-risk modifiers. Methods: We retrospectively analyzed 266 patients with acute DeBakey type I dissection treated by hemiarch repair alone (No. = 160) or hemiarch plus AMDS (No. = 106). Blinded preoperative CT-scan re-review validated Gate 1 (visible arch tear), and operative-report review confirmed Gate 2 (tear directly observed at open inspection). Supra-aortic false-lumen communication was assessed only after both gates were negative. Results: Sixty-nine patients (25.9%) passed both study-defined gates (AMDS = 32; hemiarch = 37). Twelve-month CT-scan was available in 45/69 (65.2%; AMDS 24/32, hemiarch 21/37). Among imaged patients, complete zone 2–3 false-lumen exclusion occurred in 20/24 (83.3%) after AMDS and 0/21 after hemiarch (risk difference +83.3 percentage points, 95% CI +58.7 to +93.3; *p* < 0.001). DANE occurred in 4/32 (12.5%) and 16/37 (43.2%), respectively (*p* = 0.007). Under the adverse opposing missingness scenario, the exclusion risk difference narrowed to +19.3 percentage points (95% CI −4.2 to +39.9; *p* = 0.148). Conclusions: These findings are hypothesis-generating and apply to a selected post hoc subgroup; treatment comparisons are non-causal. Prospective external validation of the study-defined hierarchy remains required.

## 1. Introduction

Acute DeBakey type I aortic dissection is a time-critical disease in which the index operation must restore proximal aortic integrity, address clinically relevant entry tears and malperfusion, and create a durable platform for the residual dissected aorta [1,2]. The contemporary arch-repair landscape spans conventional, frozen-elephant-trunk, hybrid, and endovascular strategies, reinforcing the need to match repair extent to tear location, patient condition, and institutional expertise [1,2,3].

The Ascyrus Medical Dissection Stent (AMDS) Hybrid Prosthesis is an uncovered arch scaffold deployed during open repair. Current United States labelling specifies acute DeBakey type I dissection with malperfusion, a primary entry tear in the ascending aorta proximal to the innominate artery, treatment within 0–14 days, and anatomy suitable for available device sizes; the device has not been studied when the primary tear is distal to the innominate artery [4,5]. These boundaries make tear location an applicability variable rather than merely a prognostic covariate.

Prospective and observational AMDS studies have reported high technical success, proximal true-lumen expansion, false-lumen thrombosis, and a low frequency of distal anastomotic new entry (DANE) in selected populations [6,7,8,9]. A recent two-centre descriptive series from members of our group reported clinical and radiological follow-up in 46 AMDS recipients but did not include a hemiarch comparator or test the present sequential eligibility architecture [10]. DARTS and PERSEVERE imaging analyses further linked supra-aortic false-lumen communications with less favourable downstream remodelling [11,12].

A propensity score-matched comparison found less DANE and more favourable early remodelling after hemiarch plus AMDS than after hemiarch alone, while CT-scan focused work has emphasized entry-tear location and branch-vessel involvement [13,14]. However, published evidence has not evaluated a source-adjudicated sequence in which preoperative CT-scan and direct open-arch inspection function as non-compensable, study-defined stop gates before branch communication is considered. The proposed gates are therefore an analytical framework for hypothesis generation rather than validated clinical contraindications or an established decision rule [15].

We therefore evaluated a source-adjudicated sequential anatomical eligibility architecture in a historical cohort. The primary objective was to quantify the cohort passing two study-defined arch-tear stop gates and to compare AMDS with hemiarch only among patients passing both gates. We hypothesized that an arch tear identified on preoperative CT-scan or at direct open-arch inspection would define a study-defined stop gate, whereas supra-aortic false-lumen communication would remain a residual-risk modifier after both exclusions. The hierarchy was intended to reflect the chronology and mechanics of anatomical assessment, and not to function as an additive score or a substitute for full regulatory eligibility.

## 2. Materials and Methods

### 2.1. Study Design and Population

This retrospective observational cohort analysis was prepared in accordance with the Strengthening the Reporting of Observational Studies in Epidemiology (STROBE) statement [16]. The study was conducted at our institutions. The source database contained 266 consecutive patients treated for acute DeBakey type I dissection from 2015 through 2023: 160 underwent conventional hemiarch repair and 106 underwent hemiarch repair with adjunctive AMDS (Ascyrus Medical Gmbh, Artivion Inc., Frankfurt am Main, Germany). Historical treatment allocation was non-randomized and determined by the operating team; the present eligibility architecture was specified for this retrospective re-analysis and did not determine historical care.

The study was approved by the responsible institutional review boards. Because this retrospective analysis used deidentified clinical data collected during routine care, the requirement for written informed consent was waived. The study was conducted in accordance with the Declaration of Helsinki.

### 2.2. Anatomical Variables and Sequential Eligibility Architecture

The type-entry-malperfusion framework informed anatomical terminology [17]. The hierarchy followed the chronology of information available to the surgeon. Gate 1 was positive when blinded re-review of the preoperative CT-scan confirmed a visible arch entry/tear corresponding to the database zone-2 mapping. Because CT is available before operative commitment, this finding formed the first study-defined stop gate. Among Gate-1-negative patients, Gate 2 was positive when operative-report review confirmed that the recorded primary intimal tear location of “arch” represented a tear directly observed during open-arch inspection. Gate 2 was therefore applied only when preoperative CT-scan had not already identified arch-tear anatomy.

Before final analysis, the relevant preoperative CT-scan examinations were re-read in blinded fashion, and the zone-2 visible-tear mapping was validated. Operative reports were then reviewed to verify the meaning of primary intimal tear location = arch. This source adjudication confirmed both mappings used in the final analysis. The two findings were treated as sequential, non-compensable study-defined stop gates. This hierarchy was specified on mechanistic grounds rather than derived from the Firth model: an uncovered scaffold does not itself provide a covered seal across an unresected tear. Accordingly, Gate 2 should not be interpreted as an empirically validated contraindication or as evidence that a directly inspected tear independently predicts failure. Neither gate is presented as a standalone formal contraindication in device labelling.

Only patients negative at both tear gates were classified as study-defined anatomically eligible. Within this group, any false-lumen communication involving the brachiocephalic, left common carotid, or left subclavian artery defined a high-caution phenotype, not a stop gate and not a point score. Historical AMDS recipients who did not meet one or both posthoc gates were labelled gate-discordant only for this analytical audit. Because historical treatment preceded the algorithm, this label does not imply protocol violation, inappropriate treatment, or deviation from contemporaneous practice.

### 2.3. Imaging and Outcomes

CT-scan observations were available preoperatively, before discharge, and at 3-, 12-, 24-, and 36-months follow-up at aortic zones 2, 3, 4, and 6. The prespecified primary anatomical endpoint for this re-analysis was incomplete proximal false-lumen exclusion on observed 12-month CT-scan, defined as a patent or partially thrombosed false lumen at zone 2 or 3. Complete exclusion required complete thrombosis or absence of the false lumen at both levels. Favourable proximal remodelling was the broader recorded endpoint accepting positive remodelling. Clinical endpoints were DANE, 30-day mortality, postoperative ischaemic stroke, and aortic reintervention. DANE was defined by the source imaging protocol as a new contrast-enhanced communication between the true and false lumen arising within 20 mm of the distal surgical anastomosis, directly continuous with both lumens on multiplanar computed tomography angiography (CTA) and absent at the corresponding location on preoperative imaging. Pre-existing re-entry tears and dynamic branch-point or supra-aortic fenestrations were not classified as DANE unless a new communication meeting these criteria appeared at the distal anastomosis. Pre- and postoperative CTA examinations used for DANE adjudication were re-reviewed in multiplanar reconstruction by two observers blinded to clinical outcome, with disagreements resolved by consensus. Follow-up imaging was not re-read by an external core laboratory, and formal inter-reader reliability for the broader outcome-imaging variables was not quantified.

Additional source-data review reconstructed operative-era cerebral-protection and distal-anastomotic practices and re-assessed AMDS device sizing. The operative-era extraction used an era-specific conventional/pre-AMDS denominator of 46 cases, whereas AMDS-specific device and sizing analyses used all 32 study-defined eligible AMDS recipients; these denominators describe different classification axes and are not treatment-arm denominators for the primary 37-versus-32 comparison. Cerebral perfusion mode, nadir core temperature, near-infrared spectroscopy (NIRS), electroencephalographic monitoring, and distal anastomotic reinforcement were abstracted from complete operative records. Device sizing was re-assessed from preoperative CTA using manufacturer-specific proximal and distal aortic diameter measurements rather than compressed true-lumen diameter alone. Distal stent-induced new entry (SINE) was defined as a new intimal defect at or immediately adjacent to the distal AMDS frame, absent preoperatively, with new true-to-false-lumen communication.

### 2.4. Statistical Analysis

Continuous variables are reported as median [interquartile range] and categorical variables as No.(%). Baseline imbalance was summarized using absolute standardized mean differences (SMDs), which were treated as descriptive diagnostics rather than significance tests [18]. The sequential audit reports count after each source-adjudicated gate.

Within historical AMDS recipients with observed 12-month CT-scan, two-sided Fisher exact tests evaluated univariable anatomical associations. A joint Firth penalized logistic regression model addressed sparse cells and separation and included CT-scan visible arch tear, directly inspected arch tear, and supra-aortic false-lumen communication [19]. Odds ratios (ORs) are reported with Wald 95% confidence intervals (CIs). The model was exploratory; gate coefficients were not used to define or validate the study-defined gates and were not interpreted as safety tests of gate-positive anatomy.

Within the study-defined eligible subgroup, AMDS and hemiarch outcomes were compared using two-sided Fisher exact tests. Absolute risk differences (AMDS minus hemiarch) were accompanied by Newcombe hybrid-score 95% CIs [20]. Imaging analyses used observed 12-month CT-scan denominators; missing outcome imaging was not imputed in the primary analysis.

Because the eligible cohort and several event cells were small, no high-dimensional propensity model was fitted. The subgroup was defined post hoc, treatment allocation was non-randomized, and all comparative estimates are exploratory associations rather than causal treatment effects. No multiplicity adjustment was applied; *p* values are descriptive and should be interpreted together with effect sizes and CIs. Statistical analyses were performed using IBM SPSS Statistics for Windows, Version 22.0 (IBM Corp., Armonk, NY, USA).

## 3. Results

### 3.1. Sequential Eligibility Audit

**Figure 1:**
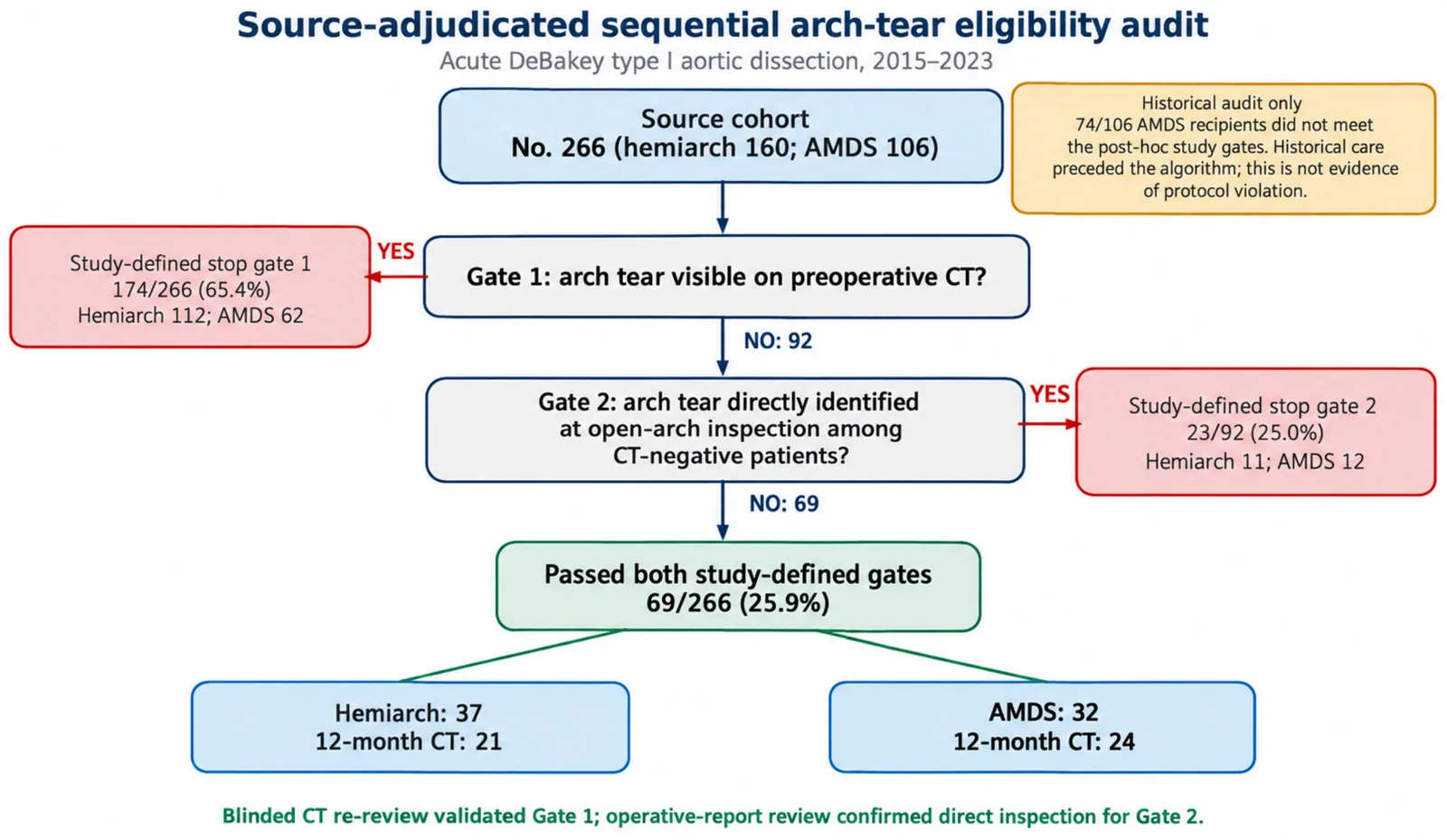
Source-adjudicated sequential eligibility audit. Gate 1 was a CT-scan visible arch tear confirmed by blinded preoperative CT-scan re-review. Among CT-scan negative patients, Gate 2 was an arch tear confirmed at direct open-arch inspection through operative-report adjudication. Historical treatment preceded the post hoc algorithm; gate discordance is not evidence of protocol violation. AMDS, Ascyrus Medical Dissection Stent; CT-scan computed tomography.

Gate 1 was positive in 174/266 patients (65.4%). Of the 92 Gate-1-negative patients, 23 (25.0%) had an arch tear confirmed at direct open-arch inspection. Thus, 69/266 patients (25.9%) passed both source-adjudicated study-defined gates; 32 AMDS recipients and 37 hemiarch controls (Figure 1). The outcome comparison therefore represents a narrow post hoc anatomical subset of the source cohort. Seventy-four of 106 AMDS recipients did not meet the post hoc study gates. Historical implantation preceded this algorithm; accordingly, the count is an anatomical audit result and is not interpreted as evidence of protocol violation.

### 3.2. Baseline Profile of the Study-Defined Eligible Cohort

The study-defined eligible groups were similar in age, sex, hypertension, and German Registry for Acute Aortic Dissection Type A (GERAADA) score, but clinically important imbalances remained (Table 1). AMDS recipients were treated later (median calendar year 2021 versus 2018; absolute SMD 1.292), and coronary malperfusion was more frequent in the AMDS group (18.8% versus 2.7%; absolute SMD 0.537). Together with non-randomized treatment allocation, these imbalances reinforce the non-causal interpretation of all treatment comparisons.

Operative-era review documented substantial secular evolution in cerebral protection and distal reconstruction (Supplementary Table S10). In the 46 conventional/pre-AMDS-era cases, unilateral antegrade cerebral perfusion (ACP) was used in 28 (60.9%), bilateral ACP in 13 (28.3%), and retrograde cerebral perfusion in 5 (10.9%); median nadir core temperature was 24.0 °C [IQR 22.0–25.0]. NIRS was used in 35/46 (76.1%), additional EEG monitoring in 9/46 (19.6%), and distal anastomotic reinforcement in 39/46 (84.8%; external felt strip in 25 and felt-neomedia/sandwich reconstruction in 14). Among all 32 eligible AMDS recipients, ACP was used in every case: unilateral in 6/32 (18.8%) and bilateral in 26/32 (81.3%), with no retrograde cerebral perfusion. Median nadir core temperature was 26.0 °C [IQR 25.0–27.0]; NIRS was used in 32/32 (100%), electroencephalogram (EEG) in 17/32 (53.1%), and distal reinforcement in 32/32 (100%), incorporating the device polytetrafluoroethylene (PTFE) cuff with external felt reinforcement. These era-specific differences reinforce the potential for secular confounding and are not interpreted as treatment effects.

### 3.3. Outcomes Within the Study-Defined Eligible Cohort

**Figure 2:**
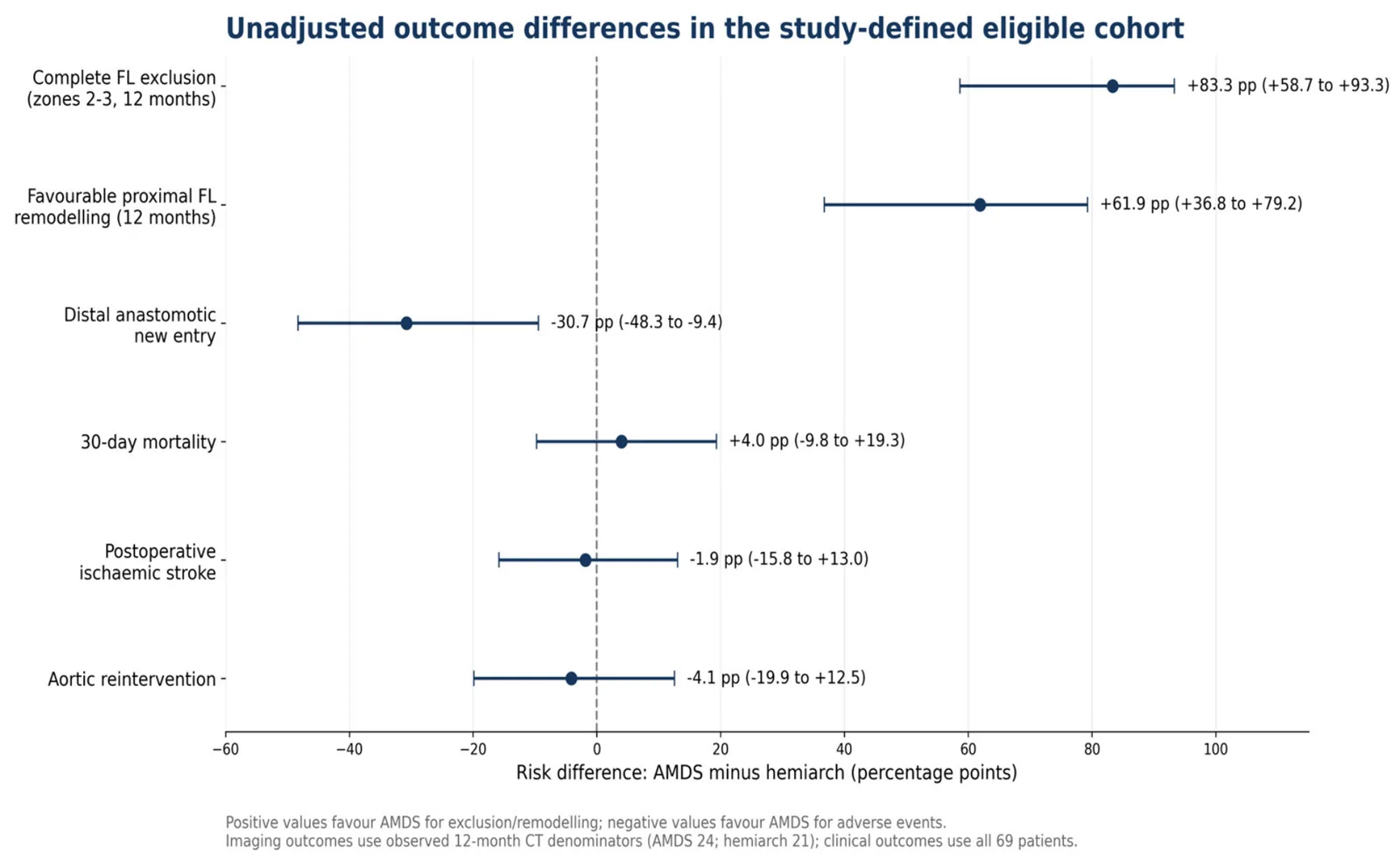
Unadjusted outcome differences in the study-defined eligible cohort. Positive values favour AMDS for exclusion/remodelling, whereas negative values favour AMDS for adverse events. Imaging outcomes use observed 12-month CT denominators; clinical outcomes use the full eligible cohort. Bars are Newcombe 95% confidence intervals. AMDS, Ascyrus Medical Dissection Stent; CI, confidence interval; DANE, distal anastomotic new entry; FL, false lumen; RD, risk difference.

Twelve-month CT-scan was available in 24/32 AMDS recipients (75.0%) and 21/37 hemiarch patients (56.8%; *p* = 0.134); thus, only 45/69 eligible patients (65.2%) contributed to the primary 12-month imaging comparison. Source-data review accounted for all 24 patients without analyzable 12-month CTA: death before scheduled imaging in 5/24 (20.8%), refusal or loss to follow-up in 6/24 (25.0%), external CT-scan with images unavailable for central analysis in 4/24 (16.7%), transfer of follow-up to a geographically distant/referring centre in 3/24 (12.5%), renal dysfunction precluding contrast-enhanced CT-scan in 4/24 (16.7%), and other documented medical reasons in 2/24 (8.3%) (Supplementary Table S7). Missingness was therefore not entirely random, and the 12-month imaging estimates should be interpreted as conditional on survival and availability of analyzable follow-up imaging. Among imaged patients, complete zone 2–3 false-lumen exclusion occurred in 20/24 (83.3%) after AMDS and 0/21 after hemiarch (risk difference +83.3 percentage points, 95% CI +58.7 to +93.3; *p* < 0.001). Favourable proximal remodelling occurred in 24/24 (100.0%) and 8/21 (38.1%), respectively (risk difference +61.9 percentage points, 95% CI +36.8 to +79.2; *p* < 0.001) (Table 2; Figure 2).

DANE occurred in 4/32 AMDS recipients (12.5%) and 16/37 hemiarch patients (43.2%; risk difference −30.7 percentage points, 95% CI −48.3 to −9.4; *p* = 0.007). Thirty-day mortality, postoperative ischaemic stroke, and aortic reintervention estimates were imprecise, with wide CIs spanning clinically important benefit and harm (Table 2; Figure 2). The three 30-day deaths in the eligible AMDS subgroup were adjudicated as persistent left-main coronary malperfusion with refractory low-output syndrome despite coronary revascularization, irreversible mesenteric malperfusion with bowel ischaemia and multiorgan failure, and severe cerebral malperfusion with a large territorial ischaemic stroke and malignant cerebral edema. None showed AMDS migration, retrograde aortic dissection, aortic rupture, or other direct device-related structural failure. The three AMDS reinterventions comprised urgent PCI for persistent right-coronary malperfusion, carotid stenting for persistent static left common carotid malperfusion, and follow-up thoracic endovascular aortic repair (TEVAR) for progressive false-lumen pressurization associated with a newly developed distal stent-induced entry at the distal AMDS edge (Supplementary Table S8). These non-randomized comparisons support anatomical plausibility but do not establish treatment superiority.

Device-model and sizing adjudication was complete for all 32 eligible AMDS recipients (Supplementary Table S11). Implanted devices were AMDS 40 straight in 6/32 (18.8%), AMDS 40–30 tapered in 6/32 (18.8%), AMDS 55 straight in 5/32 (15.6%), and AMDS 55–40 tapered in 15/32 (46.9%). Thirty of 32 patients (93.8%) fulfilled manufacturer sizing criteria at both proximal and distal measurement levels; 2 (6.2%) had a minor retrospective size mismatch, as detailed in Supplementary Table S11. Two definite distal SINE events were identified (2/32, 6.2%); both occurred in patients whose implanted device fulfilled predefined sizing criteria. One SINE was associated with progressive false-lumen pressurization/distal aortic enlargement and subsequent TEVAR. With only two SINE events and two sizing mismatches, no inferential test of a sizing-SINE association was performed.

### 3.4. Anatomical Failure Among Historical AMDS Recipients

Among 66/106 AMDS recipients with observed 12-month CT-scan, 18 (27.3%) had incomplete proximal false-lumen exclusion. Failure was more frequent with a CT-scan visible arch tear than without one (14/36 [38.9%] versus 4/30 [13.3%]; *p* = 0.027) and with a supra-aortic false-lumen communication than without one (9/12 [75.0%] versus 9/54 [16.7%]; *p* < 0.001). The directly inspected arch-tear finding alone was not associated with failure (3/11 [27.3%] versus 15/55 [27.3%]; *p* = 1.000). This small, underpowered null comparison neither validates nor refutes Gate 2, which was specified as a mechanistic study-defined stop gate rather than derived from the outcome model.

**Figure 3:**
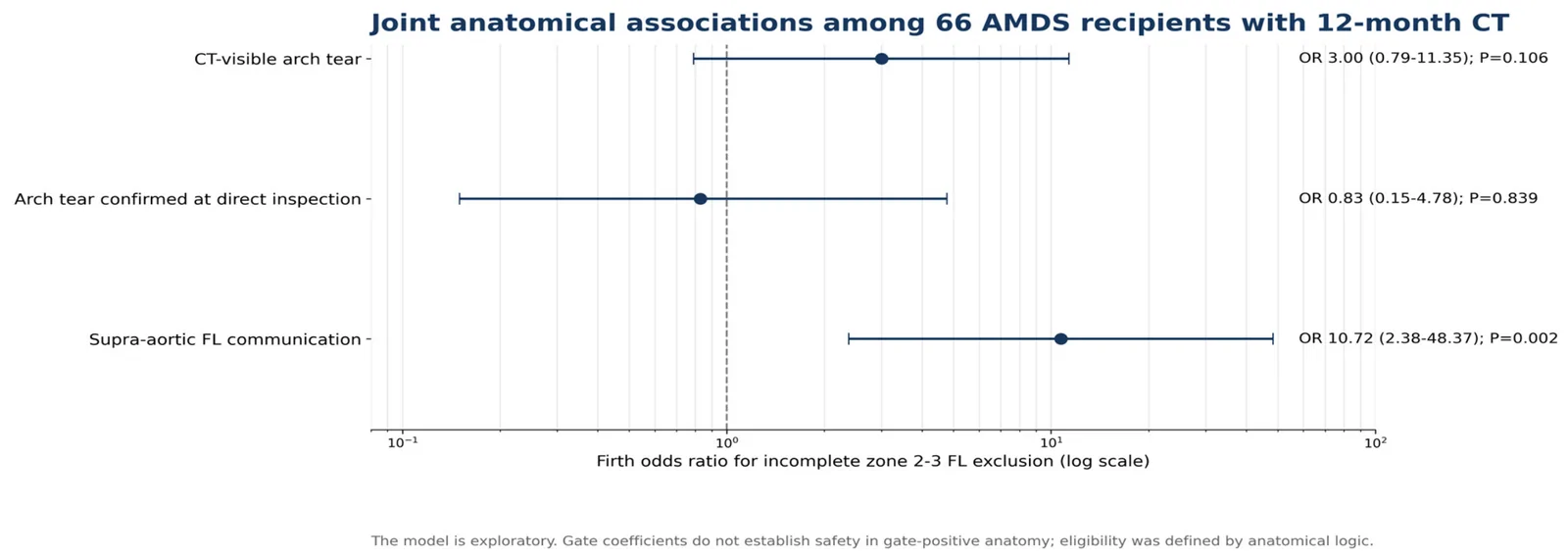
Joint Firth associations with incomplete zone 2–3 false-lumen exclusion among 66 historical AMDS recipients with observed 12-month CT-scan. Gate status was source-adjudicated. The model is exploratory, and the gate coefficients are not safety tests of arch-tear anatomy. CI, confidence interval; FL, false lumen; OR, odds ratio.

In the joint Firth model, supra-aortic communication remained associated with incomplete exclusion (OR 10.72, 95% CI 2.38–48.37; *p* = 0.002). Estimates for CT-scan visible arch tear (OR 3.00, 95% CI 0.79–11.35; *p* = 0.106) and directly inspected arch tear (OR 0.83, 95% CI 0.15–4.78; *p* = 0.839) were imprecise (Table 3; Figure 3). These coefficients describe exploratory associations within historical AMDS recipients and do not validate, override, or establish safety for either study-defined stop gate.

### 3.5. Residual-Risk Phenotype and Sensitivity Analyses

Within the 32 eligible AMDS recipients, 28 had no supra-aortic communication and 4 had at least one communication. Among those imaged at 12-months, incomplete exclusion occurred in 2/22 (9.1%) without communication and 2/2 (100.0%) with communication (*p* = 0.022). Across the full eligible cohort, 13 patients had at least one supra-aortic false-lumen communication (9 hemiarch and 4 AMDS). On branch-specific multiplanar CTA re-review, communication involved the brachiocephalic artery in 7/13 patients (53.8%), the left common carotid artery in 7/13 (53.8%), and the left subclavian artery in 6/13 (46.2%); multiple vessels could be involved in the same patient. Patient-level patterns are reported descriptively in Supplementary Table S9. Because only two high-caution AMDS patients had 12-month CT-scan and branch observations were non-independent within patients, these findings remain hypothesis-generating and no branch-specific hypothesis testing was performed.

In minimally adjusted Firth models, the direction of the AMDS association persisted for complete exclusion (OR 245.28, 95% CI 8.22–7318.88; *p* = 0.002) and DANE (OR 0.14, 95% CI 0.03–0.64; *p* = 0.012), but the exclusion estimate was extremely imprecise and both models remained vulnerable to residual confounding (Supplementary Table S3). In the calendar-overlap cohort restricted to 2018–2023 (No. = 51), observed risk differences remained +83.3 percentage points for complete exclusion and −34.9 percentage points for DANE (Table 4; Supplementary Table S4). Restriction to the overlap period reduces calendar separation but does not control unmeasured secular changes in operative technique or perioperative care.

Extreme-case analyses showed that the complete-exclusion difference remained positive when all missing CT-scans in both groups were classified uniformly as failures or uniformly as successes. Under the deliberately adverse opposing assumption that every missing AMDS CT-scan represented failure and every missing hemiarch CT-scan represented success, the risk difference narrowed to +19.3 percentage points (95% CI −4.2 to +39.9; *p* = 0.148). Therefore, the primary imaging result is robust to same-direction assumptions but not to sufficiently differential informative missingness (Supplementary Table S5). Reasons for absent 12-month CT-scans were not adjudicated in this re-analysis; accordingly, no specific missingness mechanism can be assumed.

## 4. Discussion

### 4.1. Principal Findings

This analysis proposes a decision architecture rather than another prognostic score. First, a CT-scan visible arch tear confirmed by blinded re-review defined the initial study-defined stop gate. Second, among CT-scan negative patients, operative-report adjudication confirmed whether an arch tear had been directly identified during open inspection. Third, supra-aortic false-lumen communication was interpreted only after both gates were negative. Restricting comparison to the 69 patients passing both gates produced large observed differences in proximal false-lumen exclusion and DANE, while clinical events were too sparse for reliable comparative conclusions. Because these 69 patients represent only 25.9% of the 266-patient source cohort, the comparison is subject to selection and spectrum bias and should not be generalized to the broader population with acute DeBakey type I dissection.

The distinction between eligibility and prognosis is central. In an additive score, a favourable feature can compensate numerically for a disqualifying feature. That logic is unsuitable when a finding changes the intended repair or lies outside a labelled population. Current United States labelling requires more than absence of an arch tear, including malperfusion, an ascending primary entry tear proximal to the innominate artery, a 0–14-day treatment window, and appropriate sizing [4,5]. The proposed study-defined stop gates therefore do not constitute validated clinical contraindications and cannot be used as a complete indication checklist.

### 4.2. Anatomical Mechanism and Prior Evidence

The observed pattern is anatomically plausible. AMDS is designed to stabilize the dissected arch and support true-lumen expansion while preserving flow through the supra-aortic branches [6,7,8,9,10]. Its uncovered design does not directly seal every residual communication. DARTS and PERSEVERE analyses independently linked supra-aortic false-lumen communications with less favourable downstream remodelling, supporting persistent parallel false-lumen inflow as a candidate mechanism [11,12]. The rationale for Gate 2 is likewise mechanistic: an uncovered scaffold does not provide a covered seal across a tear that remains open at direct inspection. The present regression does not empirically prove that Gate 2 predicts failure, and the nonsignificant coefficient should not be interpreted as evidence of safety in gate-positive anatomy.

The present analysis complements, rather than duplicates, the recent 46-patient descriptive series from members of our group [10]: that report focused on clinical and radiological outcomes without a hemiarch comparator, whereas this work audits sequential anatomical eligibility and restricts comparison to doubly eligible anatomy. The eligible-cohort findings are also directionally consistent with the propensity score-matched comparison by Pitts and colleagues, which reported less DANE and better early remodelling with adjunctive AMDS [13]. DANE has previously been associated with persistent false-lumen patency and adverse downstream consequences after type I dissection repair [21]. However, the strong calendar-year imbalance, non-randomized treatment allocation, coronary-malperfusion imbalance, and small sample preclude causal attribution.

Preoperative reporting should explicitly describe entry-tear location and supra-aortic branch involvement, as emphasized in recent radiologic guidance [14]. Commentary accompanying the DARTS communication analysis and the one-year PERSEVERE results has likewise highlighted the need to identify and, in selected patients, address residual sources of false-lumen pressurization [15,22]. A recent case report of head-vessel reintervention after hybrid repair further illustrates the potential clinical relevance of branch-vessel inflow, although a single case cannot establish a treatment strategy [23].

### 4.3. Proposed Prospective Workflow

**Figure 4:**
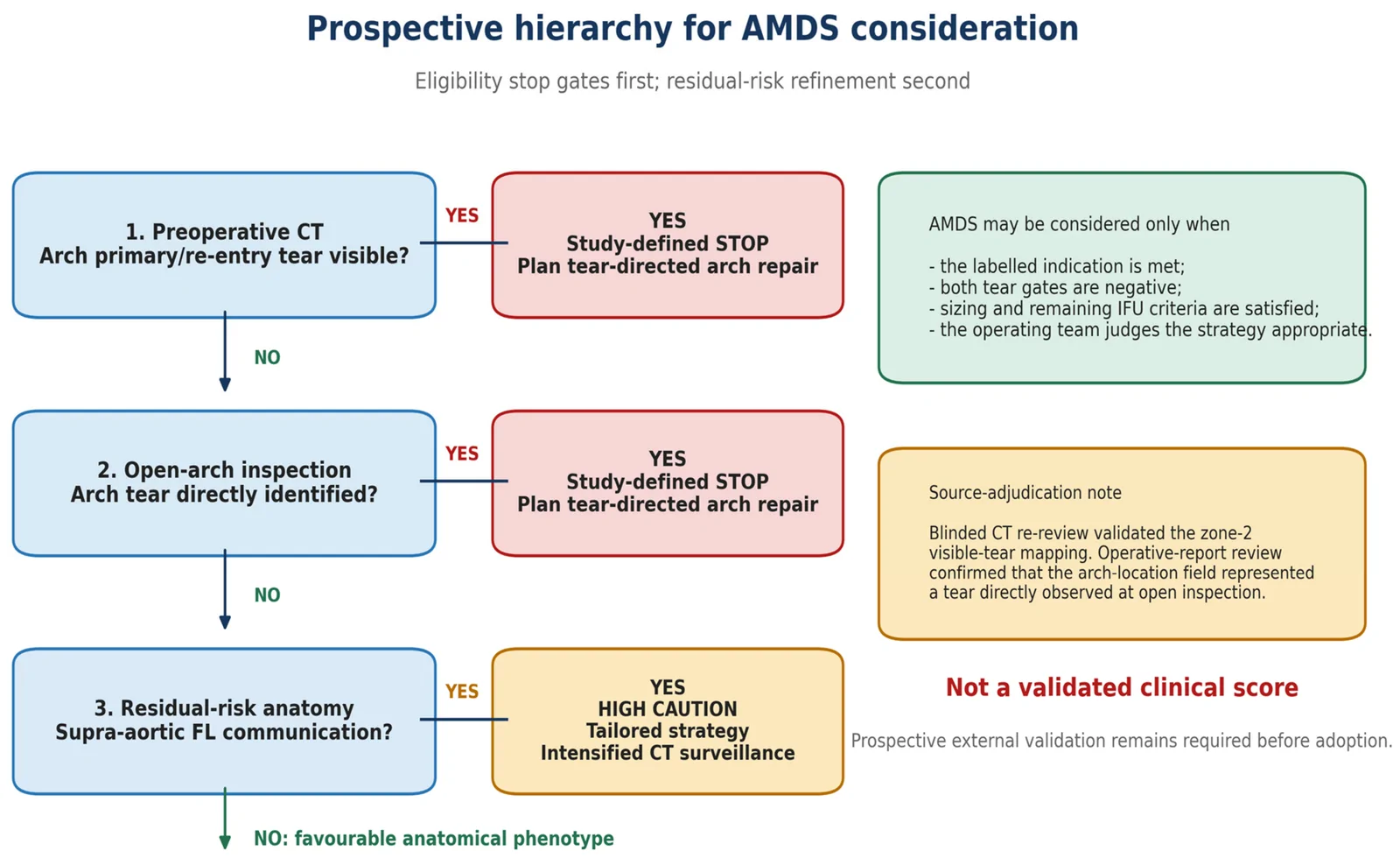
Proposed prospective hierarchy for AMDS consideration. The retrospective mappings underlying both stop gates were source-adjudicated before the final analysis: blinded CT-scan re-review validated Gate 1, and operative-report review confirmed direct open-arch identification for Gate 2. Both tear findings are study-defined stop gates. Supra-aortic false-lumen communication denotes caution only after both gates are negative. IFU, instructions for use.

A prospective version of the hierarchy would begin with a dedicated CT-scan review for arch primary or re-entry tears. If CT-scan is negative, the open arch would be inspected before committing to the final distal strategy. A visible arch tear at either stage would meet a study-defined stop gate and prompt consideration of a tear-directed repair selected according to anatomy, patient condition, labelling, and local expertise. Only after both study-defined gates were negative would supra-aortic communication refine expected remodelling and surveillance intensity (Figure 4).

### 4.4. Strengths and Limitations

Strengths include explicit separation of applicability from residual risk, blinded CT-scan re-review, operative-report adjudication, complete accounting through each gate, use of absolute effects with CIs, penalized regression for sparse data, transparent baseline SMDs, and sensitivity analyses for calendar era and missing CT-scan. The analysis also avoids using a nonsignificant gate coefficient to argue that gate-positive anatomy is safe.

The study has some limitations too. First, this study is retrospective and post-hoc; the hierarchy was not prospectively registered and requires external validation. Although blinded CT-scan re-review and operative-report review confirmed the analytical mappings, adjudication was retrospective and formal inter-reader reliability was not quantified for the broader outcome-imaging variables. Conditioning on both posthoc gates selected only 69/266 patients and may have altered the clinical spectrum of the treatment groups, limiting generalizability. The 74 AMDS recipients who did not meet the posthoc gates remain an audit subgroup only: historical care preceded the algorithm, and the finding must not be interpreted as protocol violation or inappropriate treatment.

Second, treatment allocation was non-randomized and strongly confounded by calendar era and coronary malperfusion. The 2018–2023 overlap analysis shown in the main manuscript (Table 4) reduces calendar separation but cannot eliminate secular changes. Source-data reconstruction confirmed evolution toward more bilateral ACP, warmer nadir temperature, more intensive cerebral monitoring, and more uniform distal reinforcement in the AMDS-era experience (Supplementary Table S10). These differences provide concrete evidence of historical co-intervention and reinforce that the observed AMDS-hemiarch associations cannot be attributed solely to the device. Mechanism-specific source adjudication clarified the three AMDS deaths and three reinterventions, but the small event counts and wide CIs still preclude comparative causal inference.

Third, only 45 eligible patients had analyzable 12-month CT-scans, with lower imaging availability after hemiarch repair. Source review explained all 24 missing examinations, including five deaths before scheduled imaging, demonstrating that missingness was not entirely random. The adverse opposing extreme-case scenario crossed the null; accordingly, 12-month remodelling and false-lumen exclusion estimates are conditional on survival and imaging availability rather than complete-cohort estimates. Formal inverse-probability-of-censoring weighting or missing-not-at-random multiple imputation was not performed because the available retrospective sample did not support a credibly specified multivariable missingness model; extreme-case analyses were retained as transparent stress tests. Branch-specific communication patterns, the protocol-defined DANE rule, and complete AMDS sizing/SINE adjudication are now described, but the small subgroups remain descriptive. Two definite SINE events occurred among 32 eligible AMDS recipients, both in sizing-concordant implants; this sparse event count cannot support a causal sizing analysis. Finally, the study-defined gates do not establish full regulatory eligibility and should not be extrapolated beyond the studied anatomy or labelling.

### 4.5. Future Validation

A prospective validation study should prespecify the hierarchy, use blinded central CT-scan review, record direct open-arch findings with standardized photographs or operative diagrams, distinguish primary from re-entry tears, define DANE relative to the distal suture line, quantify branch-specific communications, record device sizing and distal stent-induced new entry, and capture cerebral-perfusion and anastomotic-reinforcement strategies, mechanism-specific adverse events, reasons for missing imaging, and all labelling variables. Consecutive enrollment with complete CT-scan follow-up and external centres would permit calibration of residual-risk estimates and comparison of tear-directed strategies.

## 5. Conclusions

In this source-adjudicated retrospective anatomical audit, a hierarchical two-gate approach identified 69 of 266 patients as study-defined eligible for comparison. Within this selected posthoc subgroup, AMDS was associated with more complete proximal false-lumen exclusion and less DANE than hemiarch alone, while mortality, stroke, and reintervention estimates were inconclusive. Supra-aortic false-lumen communication identified a residual high-caution phenotype among AMDS recipients. These findings are hypothesis-generating and support prospective evaluation of sequential eligibility rather than additive scoring; they do not establish causal treatment superiority, a validated clinical rule, or generalizability to the broader DeBakey type I population.

## Figures and Tables

**Table 1:** Baseline characteristics among patients passing both source-adjudicated arch-tear gates.

Variable	Hemiarch (No. = 37)	AMDS (No. = 32)	Absolute SMD
Age, years	64.2 [57.9, 70.5]	61.0 [51.8, 70.3]	0.184
Body mass index, kg/m^2^	28.2 [27.0, 31.0]	29.4 [27.2, 34.4]	0.358
GERAADA score	13.3 [10.1, 15.9]	12.2 [9.1, 16.2]	0.100
Calendar year	2018.0 [2016.0, 2020.0]	2021.0 [2019.0, 2022.0]	1.292
Female sex	13 (35.1%)	10 (31.2%)	0.083
Hypertension	19 (51.4%)	16 (50.0%)	0.027
Chronic kidney disease	6 (16.2%)	2 (6.2%)	0.320
Preoperative tamponade	0 (0.0%)	2 (6.2%)	0.365
Preoperative CPR	1 (2.7%)	0 (0.0%)	0.236
Cerebral malperfusion	2 (5.4%)	1 (3.1%)	0.113
Coronary malperfusion	1 (2.7%)	6 (18.8%)	0.537
Renal malperfusion	3 (8.1%)	3 (9.4%)	0.045
Visceral malperfusion	0 (0.0%)	0 (0.0%)	0.000
Lower-limb malperfusion	1 (2.7%)	2 (6.2%)	0.172
Supra-aortic FL communication	9 (24.3%)	4 (12.5%)	0.309

Values are median [interquartile range] or No. (%). SMDs are descriptive; larger values indicate greater between-group imbalance. AMDS, Ascyrus Medical Dissection Stent; CPR, cardiopulmonary resuscitation; FL, false lumen; SMD, standardized mean difference.

**Table 2:** Unadjusted outcomes among patients passing both source-adjudicated arch-tear gates.

Outcome	Hemiarch	AMDS	Risk Difference, pp (95% CI)	Fisher *p*
Complete zone 2–3 FL exclusion at 12 months	0/21 (0.0%)	20/24 (83.3%)	+83.3 (+58.7 to +93.3)	<0.001
Favourable proximal FL remodelling at 12 months	8/21 (38.1%)	24/24 (100.0%)	+61.9 (+36.8 to +79.2)	<0.001
Distal anastomotic new entry	16/37 (43.2%)	4/32 (12.5%)	−30.7 (−48.3 to −9.4)	0.007
30-day mortality	2/37 (5.4%)	3/32 (9.4%)	+4.0 (−9.8 to +19.3)	0.657
Postoperative ischaemic stroke	3/37 (8.1%)	2/32 (6.2%)	−1.9 (−15.8 to +13.0)	1.000
Aortic reintervention	5/37 (13.5%)	3/32 (9.4%)	−4.1 (−19.8 to +12.5)	0.716

Risk difference is AMDS minus hemiarch. Imaging outcomes use observed 12-month CT-scan denominators; clinical outcomes use all 69 eligible patients. CI, confidence interval; DANE, distal anastomotic new entry; FL, false lumen; pp, percentage points.

**Table 3:** Anatomical associations with incomplete zone 2–3 false-lumen exclusion among 66 AMDS recipients with observed 12-month CT-scan.

Feature	Absent: Failure	Present: Failure	Fisher *p*	Joint Firth OR (95% CI)	Model *p*
CT-visible arch tear	4/30	14/36	0.027	3.00 (0.79–11.35)	0.106
Arch tear confirmed at direct inspection	15/55	3/11	1.000	0.83 (0.15–4.78)	0.839
Supra-aortic FL communication	9/54	9/12	<0.001	10.72 (2.38–48.37)	0.002

Gate status was source-adjudicated before the final analysis. Coefficients are exploratory and do not validate the study-defined gates or establish safety in gate-positive anatomy. CI, confidence interval; CT, computed tomography; FL, false lumen; OR, odds ratio.

**Table 4:** Calendar-overlap sensitivity analysis restricted to 2018–2023.

Outcome	Hemiarch	AMDS	Risk Difference, pp (95% CI)	Fisher *p*
Complete zone 2–3 FL exclusion at 12 months	0/9 (0.0%)	20/24 (83.3%)	+83.3 (+47.8 to +93.3)	<0.001
Favourable proximal FL remodelling at 12 months	3/9 (33.3%)	24/24 (100.0%)	+66.7 (+32.5 to +87.9)	<0.001
Distal anastomotic new entry	9/19 (47.4%)	4/32 (12.5%)	−34.9 (−57.1 to −9.5)	0.0087

The overlap cohort contained 51 eligible patients (19 hemiarch and 32 AMDS). Imaging outcomes use observed 12-month CT-scan denominators. Restriction to the overlap period addresses calendar separation but does not control unmeasured secular changes in operative technique or perioperative care.

## Data Availability

The deidentified data that support the findings of this study may be available from the corresponding author upon reasonable request. The data are not publicly available because of participant privacy and institutional restrictions. Access is subject to approval by the relevant institutions, applicable privacy regulations, and an appropriate data-use agreement.

## Supplementary Materials

The following supporting information can be downloaded at: https://www.mdpi.com/article/10.3390/jcdd13100496/s1, Table S1, joint Firth anatomical model; Table S2, eligible AMDS residual-risk phenotype; Table S3, treatment sensitivity models; Table S4, calendar-overlap analysis; Table S5, extreme-case analysis for missing CT-scan; Table S6, source-adjudicated operational definitions; Table S7, reasons for absent 12-month CTA; Table S8, adjudicated AMDS deaths/reinterventions; Table S9, branch-specific supra-aortic communications; Table S10, operative-era cerebral-protection and distal-reinforcement data; Table S11, AMDS device sizing and distal SINE adjudication; and a completed STROBE checklist.

## Abbreviations

AMDS, Ascyrus Medical Dissection Stent; CI, confidence interval; CT, computed tomography; DANE, distal anastomotic new entry; FL, false lumen; IFU, instructions for use; IQR, interquartile range; OR, odds ratio; SMD, standardized mean difference.
