# Supplementary material for: Sequential Arch-Tear Eligibility for Uncovered Aortic Arch Stent in Acute DeBakey Type I Aortic Dissection
Source: J Cardiovasc Dev Dis. 2026 Oct 3;13(10):496. doi: 10.3390/jcdd13100496 (PMC13705436; doi:10.3390/jcdd13100496)
Supplement: Supplementary file 1 [file jcdd-13-00496-s001.zip › jcdd-4585718-STROBE checklist.pdf]

STROBE Checklist - Cohort Study

Sequential Arch-Tear Eligibility for uncovered aortic arch stent in Acute DeBakey Type I Aortic Dissection

Locations refer to numbered sections of the accompanying JCDD manuscript. The checklist reflects the source-adjudicated final submission version.

Item	Recommendation	STROBE requirement	Manuscript location	Status/notes
1	Title and abstract	Indicate the study design and provide an informative balanced summary.	Title; Abstract	Reported; title identifies source-adjudicated retrospective eligibility audit and the abstract provides a balanced design, methods, results, and limitations summary.
2	Background/rationale	Explain scientific background and rationale.	1. Introduction	Reported with guidelines, labeling, trials, branch-communication evidence, and the selection gap.
3	Objectives	State specific objectives and prespecified hypotheses.	End of 1. Introduction	Reported; objectives and hierarchical hypothesis stated. The post-hoc nature is disclosed.
4	Study design	Present key design elements early.	2.1	Reported as retrospective observational cohort and post-hoc re-analysis.
5	Setting	Describe setting, locations, and relevant dates.	2.1	Reported; multi-centre cardiac-surgery setting, 2015-2023, with historical treatment determined by the operating team.
6	Participants	Give eligibility criteria, selection methods, and follow-up.	2.1-2.3; Fig. 1	Source cohort and study-defined eligibility flow reported. The author-supplied ethics statement confirms IRB approval and consent waiver but does not specify committee name, protocol code, or approval date.
7	Variables	Clearly define outcomes, exposures, predictors, confounders, and effect modifiers.	2.2-2.3; Table S6	Reported; eligibility gates, residual-risk phenotype, primary imaging endpoint, clinical outcomes, exposures, and covariates are operationally defined.
8	Data sources/measurement	Give sources and assessment methods for each variable.	2.2-2.3	Reported; blinded preoperative CT re-review validated Gate 1 mapping, and operative-report review confirmed direct open-arch observation for Gate 2. Follow-up outcome imaging had no external core-laboratory re-read.

Item	Recommendation	STROBE requirement	Manuscript location	Status/notes
9	Bias	Describe efforts to address bias.	2.4; 3.5; 4.4	Reported; retrospective design, treatment-era confounding, conditioning on post-hoc eligibility, sparse events, and potentially informative missing CT are explicitly addressed.
10	Study size	Explain how study size was determined.	2.1; 4.4	All available 266 patients included; no a priori power calculation. Sparse sample limitations reported.
11	Quantitative variables	Explain handling of quantitative variables.	2.4	Continuous variables summarized as medians/IQR; calendar year and GERAADA standardized in sensitivity models.
12	Statistical methods	Describe all methods, confounding control, missing data, sensitivity analyses.	2.4	Reported; descriptive summaries, SMDs, Fisher tests, Firth logistic regression, Newcombe CIs, calendar-overlap models, and extreme-case missing-CT analyses are described.
13	Participants	Report numbers at each stage and reasons for non-participation.	3.1; Fig. 1	Reported; complete sequential flow from 266 source patients through Gate 1, Gate 2, eligible cohort, treatment groups, and 12-month imaging denominators is shown in Figure 1.
14	Descriptive data	Give participant characteristics and missingness.	3.2; Table 1	Reported with SMDs. Variable-specific missingness should be rechecked in the source-controlled dataset.
15	Outcome data	Report outcome events or summary measures.	3.3-3.5; Tables 2-3	Reported with explicit denominators.
16	Main results	Give unadjusted and adjusted estimates with precision.	3.3-3.5; Tables 2-3; Tables S3-S5	Reported; causal interpretation explicitly rejected.
17	Other analyses	Report subgroup and sensitivity analyses.	3.5; Tables S2-S5	Reported and labeled exploratory.
18	Key results	Summarize with reference to objectives.	4.1	Reported.
19	Limitations	Discuss limitations, direction, and magnitude of potential bias.	4.4	Reported in depth; limitations include retrospective post-hoc design, calendar-era confounding, small cells, missing imaging, no outcome core laboratory, no formal inter-reader reliability, residual confounding, and lack of external validation.
20	Interpretation	Give cautious overall interpretation.	4.1-4.5; 5	Reported with consistency and divergence from prior evidence and explicit validation requirements.
21	Generalisability	Discuss external validity.	4.4-4.5	Reported; generalizability is limited to the studied anatomy and context. Source adjudication strengthens internal classification but does not substitute for prospective multicentre validation.

Item	Recommendation	STROBE requirement	Manuscript location	Status/notes
22	Funding	State funding and funder role.	Funding; Conflicts of Interest	Reported; the authors received no specific funding, the study had no sponsor, and no funder role existed.
