# Supplementary material for: Sequential Arch-Tear Eligibility for Uncovered Aortic Arch Stent in Acute DeBakey Type I Aortic Dissection
Source: J Cardiovasc Dev Dis. 2026 Oct 3;13(10):496. doi: 10.3390/jcdd13100496 (PMC13705436; doi:10.3390/jcdd13100496)
Supplement: Supplementary file 1 [file jcdd-13-00496-s001.zip › jcdd-4585718-supplementary-1.pdf]

Supplementary Materials

Sequential Arch-Tear Eligibility for uncovered aortic arch stent in Acute DeBakey Type I Aortic Dissection

Supplementary Methods

The supplied workbook was read from the AMDS Eligibility Patients and AMDS Cohort Expanded worksheets. Automated checks confirmed 266 unique rows, QC status "OK" for all rows, and 69 patients passing both source-adjudicated gates. Blinded CT re-review validated the zone-2 visible-tear mapping.

Treatment sensitivity models were deliberately parsimonious because of sparse events. Each model included treatment alone or treatment plus standardized calendar year and standardized GERAADA score. Calendar-overlap analyses were restricted to years in which both strategies were represented (2018-2023). Extreme-case analyses classified missing 12-month CT outcomes under uniform and opposing assumptions.

Table S1. Joint Firth model for incomplete zone 2-3 false-lumen exclusion among 66 AMDS recipients with observed 12-month CT

Term	OR	95% CI lower	95% CI upper	p
Intercept	0.1187	0.0365	0.3859	0.0004
CT-visible arch tear	2.9964	0.7908	11.3546	0.1064
Arch tear confirmed at direct inspection	0.8340	0.1456	4.7787	0.8386
Supra-aortic FL communication	10.7222	2.3770	48.3653	0.0020

The model converged in 10 iterations. Wald intervals are shown. Gate coefficients do not determine anatomical eligibility or establish safety in gate-positive anatomy.

Table S2. Residual supra-aortic communication phenotype among study-defined eligible historical AMDS recipients

Eligible AMDS phenotype	n	12-month CT	Incomplete exclusion	DANE	Reintervention	30-day mortality
No supra-aortic FL communication	28	22	2/22 (9.1%)	3/28 (10.7%)	1/28 (3.6%)	2/28 (7.1%)
Supra-aortic FL communication	4	2	2/2 (100.0%)	1/4 (25.0%)	2/4 (50.0%)	1/4 (25.0%)

Fisher exact p for incomplete exclusion = 0.022. Only two high-caution patients had 12-month CT.

Table S3. Firth treatment sensitivity models in the study-defined eligible cohort

Outcome	Model	N	Term	OR	95% CI	p	Converged
Complete zone 2-3 FL exclusion	Treatment only	45	Intercept	0.02326	0.001319-0.4101	0.0102	Yes
Complete zone 2-3 FL exclusion	Treatment only	45	AMDS	195.9	9.25-4148	0.0007	Yes
Complete zone 2-3 FL exclusion	Minimally adjusted	45	Intercept	0.023	0.001545-0.3425	0.0062	Yes
Complete zone 2-3 FL exclusion	Minimally adjusted	45	AMDS	245.3	8.22-7319	0.0015	Yes
Complete zone 2-3 FL exclusion	Minimally adjusted	45	calendar_year_z	0.8879	0.2081-3.789	0.8725	Yes
Complete zone 2-3 FL exclusion	Minimally adjusted	45	geraada_z	0.396	0.1435-1.093	0.0737	Yes
Distal anastomotic new entry	Treatment only	69	Intercept	0.7674	0.4006-1.47	0.4249	Yes
Distal anastomotic new entry	Treatment only	69	AMDS	0.2057	0.06192-0.6836	0.0099	Yes
Distal anastomotic new entry	Minimally adjusted	69	Intercept	0.7893	0.3632-1.715	0.5502	Yes
Distal anastomotic new entry	Minimally adjusted	69	AMDS	0.1398	0.03038-0.6436	0.0115	Yes
Distal anastomotic new entry	Minimally adjusted	69	calendar_year_z	1.183	0.6072-2.305	0.6214	Yes
Distal anastomotic new entry	Minimally adjusted	69	geraada_z	2.444	1.289-4.633	0.0062	Yes

The complete-exclusion treatment estimate is very large and imprecise because no hemiarch patient achieved complete exclusion in the observed imaging set.

Table S4. Calendar-overlap analysis restricted to 2018-2023

Outcome	Hemiarch	AMDS	Risk difference, pp (95% CI)	Fisher p
Complete zone 2-3 FL exclusion at 12 months	0/9 (0.0%)	20/24 (83.3%)	+83.3 (+47.8 to +93.3)	<0.001
Favourable proximal FL remodelling at 12 months	3/9 (33.3%)	24/24 (100.0%)	+66.7 (+32.5 to +87.9)	<0.001
Distal anastomotic new entry	9/19 (47.4%)	4/32 (12.5%)	-34.9 (-57.1 to -9.5)	0.0087

The overlap cohort contained 51 eligible patients: 19 hemiarch and 32 AMDS.

Table S5. Extreme-case analysis for missing 12-month CT and complete zone 2-3 false-lumen exclusion

Scenario	Hemiarch complete	AMDS complete	Risk difference, pp (95% CI)	Fisher p
Observed-case analysis	0/21 (0.0%)	20/24 (83.3%)	+83.3 (+58.7 to +93.3)	<0.001
All missing in both groups classified as failure	0/37 (0.0%)	20/32 (62.5%)	+62.5 (+42.9 to +77.1)	<0.001
All missing in both groups classified as success	16/37 (43.2%)	28/32 (87.5%)	+44.3 (+22.0 to +60.7)	<0.001
Adverse opposing extremes: AMDS missing=failure; hemiarch missing=success	16/37 (43.2%)	20/32 (62.5%)	+19.3 (-4.2 to +39.9)	0.1484
Favourable opposing extremes: AMDS missing=success; hemiarch missing=failure	0/37 (0.0%)	28/32 (87.5%)	+87.5 (+69.3 to +95.0)	<0.001

Opposing extremes are deliberately implausible stress tests, not estimates of the missing outcomes. Source-data review subsequently identified the documented reason for every absent 12-month CTA; these reasons are summarized in Table S7 and confirm that missingness was not entirely random.

Table S6. Source-adjudicated operational definitions and interpretive guardrails

Term	Operational definition	Interpretation
Gate 1	Visible zone-2 arch entry/tear on preoperative CT, confirmed by blinded re-review	Source-adjudicated study-defined stop gate
Gate 2	Arch primary tear directly observed during open-arch inspection among Gate-1-negative patients, confirmed through operative-report review	Source-adjudicated study-defined stop gate specified on mechanistic grounds; not an empirically validated contraindication
Study-defined eligible	Negative at both arch-tear gates	Enters comparative and residual-risk analyses; not synonymous with full regulatory eligibility
High caution	Study-defined eligible plus any supra-aortic FL communication	Residual-risk feature; not a score or validated contraindication; branch-specific effects were not separately estimated in this re-analysis
Incomplete proximal exclusion	Patent or partially thrombosed FL at zone 2 or 3 on observed 12-month CT	Primary anatomical failure endpoint
Historical gate discordance	Historical AMDS implantation followed by a positive post-hoc Gate 1 or Gate 2	Historical treatment preceded the algorithm and this is not evidence of protocol violation

Table S7. Documented reasons for absent analyzable 12-month CTA among the 24 study-defined eligible patients without imaging

Reason	n	% of missing CTA
Death before scheduled 12-month imaging	5	20.8
Refusal or loss to follow-up	6	25.0

External CT performed; images unavailable for central analysis	4	16.7
Follow-up transferred to geographically distant/referring center	3	12.5
Renal dysfunction precluding contrast-enhanced CT	4	16.7
Other documented medical reasons preventing scheduled CTA	2	8.3
Total	24	100.0

All 24 missing examinations were explained after source-data review. Because five patients died before scheduled imaging and other reasons could also relate to clinical status or follow-up structure, 12-month imaging availability was not assumed to be missing completely at random.

Table S8. Adjudicated 30-day deaths and aortic reinterventions in the study-defined eligible AMDS subgroup

Event	Timing	Adjudicated mechanism	Device-related structural finding
30-day death 1	Early	Persistent left-main coronary malperfusion with refractory low-output syndrome despite surgical coronary revascularization	No migration, retrograde dissection, rupture, or distal SINE
30-day death 2	Early	Irreversible mesenteric malperfusion with bowel ischaemia and multiorgan failure	No migration, retrograde dissection, rupture, or distal SINE
30-day death 3	Early	Severe cerebral malperfusion with large territorial ischaemic stroke and malignant cerebral oedema	No migration, retrograde dissection, rupture, or distal SINE
Reintervention 1	Urgent postoperative	PCI for persistent right-coronary malperfusion after proximal aortic repair	No migration or rupture
Reintervention 2	Postoperative	Carotid stenting for persistent static left common carotid malperfusion	No migration or rupture
Reintervention 3	Follow-up	TEVAR for progressive false-lumen pressurization and distal aortic enlargement associated with a newly developed distal stent-induced entry at the distal AMDS edge	Definite distal SINE

Two reinterventions treated persistent malperfusion attributable to the index dissection; one later reintervention was associated with a new distal stent-related entry. No device migration, retrograde type A dissection, or aortic rupture was identified during observed follow-up.

Table S9. Branch-specific supra-aortic false-lumen communications among the 13 affected study-defined eligible patients

Patient-level communication pattern	n
Isolated brachiocephalic artery	3
Isolated left common carotid artery	2
Isolated left subclavian artery	2
Brachiocephalic + left common carotid	2
Brachiocephalic + left subclavian	1
Left common carotid + left subclavian	2
All three supra-aortic vessels	1
Total affected patients	13

Vessel-level totals were: brachiocephalic artery 7/13 (53.8%), left common carotid artery 7/13 (53.8%), and left subclavian artery 6/13 (46.2%), for 20 vessel-level communications across 13 patients. Frequencies are not mutually exclusive; no branch-specific hypothesis testing was performed.

Table S10. Operative-era cerebral protection, monitoring, and distal anastomotic reinforcement

Variable	Conventional/pre-AMDS era (n=46)	Eligible AMDS recipients (n=32)
Unilateral ACP	28/46 (60.9%)	6/32 (18.8%)
Bilateral ACP	13/46 (28.3%)	26/32 (81.3%)
Retrograde cerebral perfusion	5/46 (10.9%)	0/32 (0.0%)
Nadir core temperature, °C	24.0 [22.0-25.0]	26.0 [25.0-27.0]
NIRS monitoring	35/46 (76.1%)	32/32 (100%)
Additional EEG monitoring	9/46 (19.6%)	17/32 (53.1%)
Any distal anastomotic reinforcement	39/46 (84.8%)	32/32 (100%)
External felt strip	25/46 (54.3%)	—
Felt-neomedia/sandwich reconstruction	14/46 (30.4%)	—
PTFE cuff + external felt reinforcement	—	32/32 (100%)

The 46-case conventional/pre-AMDS-era denominator is an era-specific operative stratum, whereas the 32-case AMDS denominator is treatment-specific; these descriptors are not the mutually exclusive 37-versus-32 treatment arms used for the primary eligible-cohort comparison. ACP, antegrade cerebral perfusion; EEG, electroencephalography; NIRS, near-infrared spectroscopy; PTFE, polytetrafluoroethylene.

Table S11. AMDS device model, sizing eligibility, and distal stent-induced new entry among 32 eligible AMDS recipients

Characteristic	n/N (%) or detail

AMDS 40 straight	6/32 (18.8%)
AMDS 40-30 tapered	6/32 (18.8%)
AMDS 55 straight	5/32 (15.6%)
AMDS 55-40 tapered	15/32 (46.9%)
Manufacturer sizing criteria fulfilled proximally and distally	30/32 (93.8%)
Minor retrospective size mismatch	2/32 (6.2%)
Mismatch 1	AMDS 55-40; proximal aortic diameter 35 mm, immediately below recommended proximal interval
Mismatch 2	AMDS 40-30; distal diameter 25 mm, immediately above recommended distal range
Definite distal SINE	2/32 (6.2%)
SINE in sizing-concordant implant	2/2 (100%)
SINE requiring TEVAR	1/2; progressive false-lumen pressurization and distal aortic enlargement

Distal SINE was defined as a new intimal defect at or immediately adjacent to the distal AMDS frame, absent on preoperative imaging, with new true-to-false-lumen communication. No statistical association between sizing and SINE was tested because there were only two SINE events and two sizing mismatches. Both SINE events occurred in sizing-concordant implants; the clinical course of the second SINE was unknown.
